# Relationship of obstructive sleep apnea with periodontal condition and its local and systemic risk factors

**DOI:** 10.1007/s00784-023-04869-8

**Published:** 2023-02-17

**Authors:** Natalia Arango Jimenez, Darena Z. Morales Vera, Catalina Latorre Uriza, Juliana Velosa-Porras, Mayra A. Téllez Corral, Francina Maria Escobar Arregocés

**Affiliations:** 1 Periodontics, Faculty of Dentistry, Pontificia Universidad Javeriana, Bogotá, DC Colombia; 2 Centro de Investigaciones Odontológicas, Faculty of Dentistry, Pontificia Universidad Javeriana, Carrera 7 # 40-62, Bogotá, DC Colombia

**Keywords:** Obstructive sleep apnea (OSA), OSAHS, Periodontal disease, Periodontitis, Risk factors

## Abstract

**Objective:**

Obstructive sleep apnea (OSA) and periodontitis share risk factors, such as age, obesity, stress, and cardiovascular events, which have a bidirectional cause-effect relationship through systemic inflammation. Our objective was to determine the relationship between OSA and the periodontal condition and its associated local and systemic risk factors.

**Material and methods:**

This was an observational case-control study involving 60 patients. Local oral risk factors and the systemic condition of each patient were evaluated. All patients underwent polysomnography for the diagnosis of OSA. Chi-squared, one-way ANOVA, and Bonferroni’s tests were performed.

**Results:**

A higher percentage of patients with periodontitis had severe OSA (66.66%); however, no statistically significant association was found between the two pathologies (*p* = 0.290). In terms of systemic risk factors, an association was found between arterial hypertension and severe OSA (*p* = 0.038), and in terms of local factors, an association was found between the use of removable prostheses and severe OSA (*p* = 0.0273).

**Conclusion:**

In the general population, patients with periodontitis showed a higher prevalence of severe OSA. Obesity and hypothyroidism were the most prevalent systemic findings in patients with OSA and periodontitis. Arterial hypertension and osteoarthritis were found to be associated with severe OSA. The local risk factors associated with periodontitis and severe OSA were removable partial dentures and misfit resins.

**Clinical relevance:**

To study the factors that can facilitate the progression of OSA and periodontitis, physicians and dentists should be advised to provide comprehensive care for patients with both pathologies.

## Introduction

In 1999, the American Academy of Sleep Medicine (AASM) defined obstructive sleep apnea-hypopnea syndrome (OSAHS) as a disorder in which there is a complete or partial obstruction of the upper airway by a pharyngeal collapse with a reduction in the inspiratory airflow for at least 10 s (hypopnea) or the complete absence of it (apnea) [1]. This disorder is a relevant morbidity problem, affecting between 5 and 15% of the global population [2]. It has also been reported that the presence of OSA is associated with an increased risk of morbidity and mortality due to the decrease in oxygen supply that causes damage to the cells of the organs [3]. Therefore, OSA has been linked to systemic diseases such as hypertension, stroke, acute myocardial infarction, congestive heart failure, diabetes mellitus, depression, and excessive daytime sleepiness [4, 5]. All these systemic diseases have been associated with oral manifestations, such as periodontal disease [6].

Periodontitis is the result of a disturbance of the subgingival biofilm with concomitant alteration of its functional properties in relation to the surveillance of innate defenses and tissue maintenance, leading to excessive and dysregulated inflammation and tissue destruction. The composition of the oral microbiome is shaped throughout life by factors including host genetics, maternal transmission, as well as environmental factors, such as dietary habits, oral hygiene practice, medications, and systemic factors [7, 8]. The oral microbiota of patients with obstructive sleep apnea (OSA) and its association with periodontal disease have recently been analyzed. Periodontal pathogenic bacteria of the orange complex, such as *Prevotella melaninogenica* and the yeast *Candida albicans*, altered the culturable oral microbiota of patients with periodontitis and OSA in terms of diversity, possibly increasing the severity of the periodontal disease [9].

This inflammation in periodontal tissues results in the release of circulating cytokines, including tumor necrosis factor alpha (TNF-α), interleukin-1 beta (IL1β), interleukin-6 (IL-6), nuclear kappa beta (NF-Kβ), the ligand of the receptor activator of nuclear factor κB (RANK-L), prostaglandin E2 (PGE2), and C-reactive protein (CRP). Local and systemic inflammation seems to be the mechanism that relates periodontal disease with different pathologies, such as diabetes, cardiovascular disease, metabolic syndrome, low birth weight, osteoporosis, and, more recently, sleep apnea. In addition, evidence continues to emerge documenting the importance of oral care in the prevention of hospital-associated non-ventilator-associated pneumonia. Reducing oral biofilm in these populations will reduce the number of potential respiratory pathogens in oral secretions that can be aspirated, which in turn may reduce the risk of pneumonia [10–13].

The relationship between OSA and periodontitis was mentioned for the first time by Gunaratnam et al. [11], who reported that periodontitis was more prevalent in 77% of patients with OSA, finding a relationship four times higher than the Australian national averages. On the other hand, in a more recent study, Gamsiz-Isik et al. [13] reported that in patients with OSA, the prevalence of periodontitis was 94.6%. Based on this hypothesis, several studies have suggested that the relationship between these two diseases is given by several factors; among them, OSA is described as a hypoxic environment in multiple sites of the aerodigestive tract. Therefore, it is feasible that the oral biofilm is formed in an environment that favors the proliferation of facultative and strict anaerobic bacteria [14], especially microorganisms called periodontopathogens. It has also been proposed that the level of oxygenation in OSA modifies the composition of the human microbiota and its interactions with the host, such as alterations in the permeability of the digestive epithelium [15].

OSA and periodontitis have coincident risk factors, such as age, smoking, diabetes mellitus, obesity, stress, and cerebrovascular events. Because the relationship has not been clearly established, studying the factors that can facilitate the progression of these pathologies continues to be of interest to the scientific community. Therefore, the objective of this study was to determine the relationship between OSA and the presence or absence of periodontal disease and its associated local and systemic risk factors.

## Materials and methods

This was an observational case-control study. Sixty patients examined from April 2019 to March 2020 were recruited and divided into the following 4 groups: 15 patients without OSA and without periodontitis (H), 15 patients without OSA with periodontitis (P), 15 patients with OSA without periodontitis (O), and 15 patients with OSA and with periodontitis (OP). All were diagnosed by polysomnography (PSG). From the PSG study, the apnea-hypopnea index (AHI), calculated by dividing the number of events by the number of hours of sleep, was obtained. According to its severity, OSA was classified as mild OSA with an AHI> 5 and <15, moderate OSA with an AHI> 15 and <30, and severe OSA with an AHI> 30/hour [16].

The inclusion criteria were as follows: patients older than 30 years with at least 6 teeth in the mouth and polysomnography examination no longer than 6 months. The exclusion criteria were as follows: patients who received periodontal therapy in the last 3 months, smokers, diabetics, those who took antibiotics in the last 3 months, patients treated with CPAP or BPAP or who were under pharmacological treatment and/or who had received surgical treatment for sleep apnea.

This research is classified as minimal risk according to resolution number 8430 from 1993 of the Colombian Ministry of Health with prior approval of the Research and Ethics Committee of the Faculty of Dentistry of the Pontificia Universidad Javeriana with virtual act no. 016B. All participants signed informed consent after explaining the conditions and exams to be performed. The informed consent obtained from the study participants was written.

The clinical history included anamnesis, intraoral and extraoral examination, and recording of local and systemic risk factors. To determine the systemic factors, the following information was obtained: weight and height for calculation of body mass index (BMI), history of arterial hypertension (AHT), rheumatoid arthritis, diabetes, smoking, heart disease, immunosuppression, blood dyscrasias, hypothyroidism, chronic rhinitis, hyperlipidemia, osteoporosis, and pharmacological history. The following local oral factors were recorded: biofilm with the Silness and Loe index, as well as the number of teeth present and absent, the presence of removable partial dentures, fixed partial dentures, fillings such as resins, amalgams, and sealants, the presence of supra- or subgingival calculi, the presence of orthodontic appliances, fixed retainers, dental malposition (tooth rotations or translations), excessive occlusal forces and open interproximal contacts, wear facets, occlusal trauma, open bite, crossbite, presence of premature contacts, and occlusal interferences.

The periodontal examination was performed by two calibrated evaluators who used a North Carolina probe (Hu-Friedy®). The periodontal diagnosis was performed according to the Caton 2018 classification. In this classification, stages I to IV of periodontitis are defined based on severity (primarily periodontal breakdown with reference to root length and periodontitis-associated tooth loss) and complexity of management (pocket depth, infrabony defects, furcation involvement, tooth hypermobility, masticatory dysfunction), and additionally described as extent (localized or generalized). The grade of periodontitis is estimated with direct or indirect evidence of progression rate in three categories: slow, moderate, and rapid progression (grades A, B, C). Risk factor analysis is used as a grade modifier [17]. The periodontal pocket, the location of the gingival margin, and the level of attachment were evaluated.

### Statistical analysis

Descriptive statistics were performed with means, medians, standard deviations, and ranges, calculating the proportion of patients with OSA and periodontal disease with the local factors examined. Subsequently, to determine the association between OSA and periodontal disease, an inferential statistic was calculated by means of the chi-square test, one-way ANOVA, and Bonferroni, and a value of *p* <0.05 indicated statistical significance.

## Results

Sixty patients, 30 women and 30 men, were evaluated. The overall average age was 49.4 years (95% CI (SD 46.26–52.59)). The average age for each of the groups was 47.53 (SD 40.59–54.47) for group H, 43.06 (36.73–49.39) for group P, 53.93 (48.41–59.45) for group O, and 53.2 (47.95–58.44) for group OP.

When evaluating the diagnosis of OSA according to its severity, 18.33% presented with mild OSA, 11.67% with moderate OSA, and 20% with severe OSA, with the latter being the most prevalent condition. Regarding the periodontal condition, it was evident that 70% had stage III periodontitis.

### Periodontal clinical findings

Regarding periodontal bleeding and the presence or absence of OSA, greater bleeding was found in patients with periodontitis without OSA (81.8%) followed by patients with periodontitis with OSA (67.1%). It was observed that there was a tendency of greater bleeding on probing in the group with apnea than in the group without apnea, although the difference was not statistically significant (Table 1).

**Table 1:** Periodontal parameters in the four groups of patients

Periodontal parameters	Healthy (H)	Non-OSA with periodontitis (P)	OSA without periodontitis (O)	OSA with periodontitis (OP)	*p* value*
Bleeding on probing (%)	20.4 ± 24.8	81.8 ± 21.7†	44.3 ± 35.5†	67.1 ± 25.6†	0.0000
Gingival recession (mm)	−1.3 ± 0.7	−1.4 ± 0.5	−1.7 ± 0.7	−1.4 ± 0.3	0.3561
Biofilm (%)	23.3 ± 13.6	48.7 ± 25.3‡	35.9 ± 21.7	38.7 ± 15.8	0.0091
Probing pocket depth					
<4mm	1.7 ± 0.4	2.6 ± 0.3§	2 ± 0.1§	2.6 ± 0.4§	0.0000
≥4mm	0	4.3 ±0.5	0	4.4 ± 0.4	0.0000
CAL (mm)	1.2 ±0.6	2.2 ±0.9¤	1.6 ± 0.9	2.1 ± 0.9¤	0.0145

*H* group of healthy patients without OSA and without periodontitis, *P* group of patients with non-OSA with periodontitis, *O* group of patients with OSA without periodontitis, *OP* group of patients with OSA and with periodontitis, *CAL* clinical attachment level. *One-way ANOVA; post hoc test: multiple comparisons using Bonferroni’s comparisons test: †Bonferroni’s H vs P (*p*=0.000), P vs O (*p*=0.003), H vs OP (*p*=0.000); ‡Bonferroni’s H vs P (*p*=0.005); §Bonferroni’s H vs P (*p*=0.000), P vs O (*p*=0.001), H vs OP (*p*=0.000), O vs OP (*p*=0.000); ¤Bonferroni’s H vs P (*p*=0.026), H vs OP (*p*=0.050)

When analyzing the percentage of biofilm, it was found that in the group with periodontitis with and without apnea, the percentages were higher (38.7% and 48.7%, respectively) (Table 1).

In the evaluation of probing depth, a greater probing depth was found in the group with periodontitis, with statistically significant differences (*p* = 0.000). When the patients in the P and OP groups were analyzed, it was found that the percentage of teeth with periodontitis was 42.8% in the P group and 33.4% in the OP group. However, this difference was not statistically significant (*p* = 0.596) (Table 1).

With respect to the analysis of the clinical attachment level (CAL), it was found that there were statistically significant differences between the four study groups (*p* = 0.0145), showing a greater attachment loss in those patients with periodontitis (Table 1).

When the association between patients who presented with OSA and the periodontal condition was evaluated, it was found that of those diagnosed with periodontitis, 27.2% had mild OSA, 57.14% had moderate OSA, and 66.66% had severe OSA. Although no statistically significant association was found between the two pathologies (*p* = 0.290), there was a tendency for patients with periodontitis to have severe apnea (Table 2).

**Table 2:** Frequency and percentage of periodontal condition according to obstructive sleep apnea diagnosis

Periodontal diagnosis	OSA diagnosis								
	No OSA		Mild OSA		Moderate OSA		Severe OSA		*P* value
	Fr	%	Fr	%	Fr	%	Fr	%	
Without periodontitis	15	50	8	72.7	3	42.85	4	33.33	0.290
Periodontitis	15	50	3	27.2	4	57.14	8	66.66	
Total	30	100	11	100	7	100	12	100	

*Fr* frequency, *OSA* obstructive sleep apnea

### Systemic risk factors

**Fig. 1:**
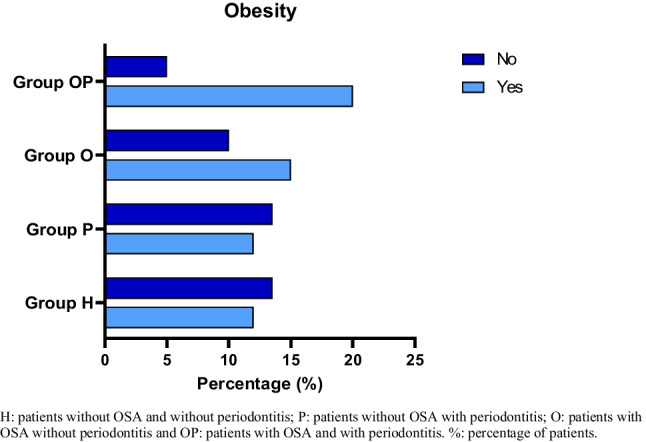
Distribution of obese and non-obese patients in all the groups. H, patients without OSA and without periodontitis; P, patients without OSA with periodontitis; O, patients with OSA without periodontitis; and OP, patients with OSA and with periodontitis. %, percentage of patients

**Fig. 2:**
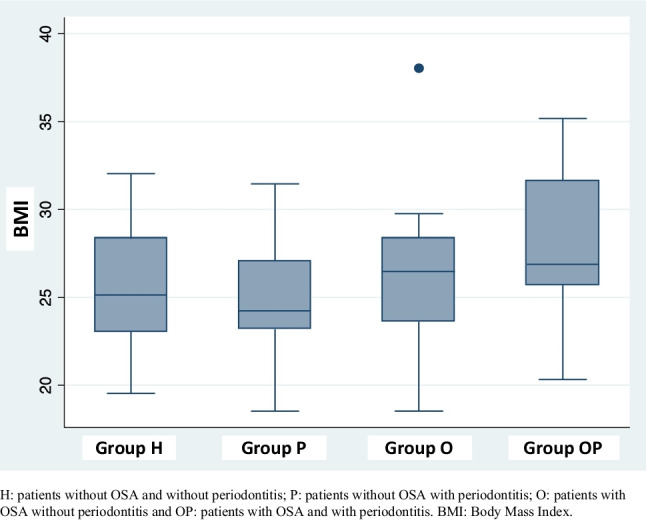
Distribution of the body mass index (BMI) of the patients in all the groups. H, patients without OSA and without periodontitis; P, patients without OSA with periodontitis; O, patients with OSA without periodontitis; and OP, patients with OSA and with periodontitis. BMI, body mass index

With respect to the systemic risk factors evaluated, it was found that one of the most frequent diseases was obesity, at 58.3%. The highest percentage of obese patients was found in the group with OSA and periodontitis (20%) (Fig. 1). Regarding BMI in the general population, an average of 26.12 kg/m^2^ was found, being higher in the OSA group with periodontitis (28.04 kg/m^2^) followed by the OSA group without periodontitis (26.31 kg/m^2^), and although the difference was not statistically significant, the trend suggests that patients who suffer from OSA have a higher BMI (Fig. 2).

The second most commonly reported systemic condition was arterial hypertension (28.3%), followed by hypothyroidism (16.6%). Additionally, other diseases, namely, hyperlipidemia, chronic rhinitis, rheumatoid arthritis, and osteoarthritis, were identified in 8.3%, 5%, 3.3%, and 3% of patients, respectively.

In the comparison of the OSA condition with the aforementioned pathologies and the presence of periodontitis, it was observed that patients with severe OSA without periodontitis had significantly more arterial hypertension than patients with severe OSA with periodontitis (*p* = 0.038). Similarly, it was shown that patients with severe OSA without periodontitis reported osteoarthritis; conversely, patients with severe OSA and periodontitis did not present with osteoarthritis, and the difference was statistically significant (*p* = 0.028) (Table 3).

**Table 3:** Frequency of systemic risk factors associated with the periodontal condition and obstructive sleep apnea diagnosis

Systemic risk factors	Without periodontitis (*n*=15)	Periodontitis (*n*=15)	*P* value
Arterial hypertension			
No OSA	3	4	0.666
Mild OSA	2	0	0.338
Moderate OSA	1	0	0.212
Severe OSA	4	3	0.038*
Cardiovascular diseases			
No OSA	0	1	0.309
Mild OSA	1	0	0.521
Moderate OSA	1	1	0.809
Rheumatoid arthritis			
No OSA	0	1	0.309
Severe OSA	0	1	0.460
Hypothyroidism			
No OSA	2	3	0.624
Mild OSA	1	2	0.072
Severe OSA	0	2	0.273
Chronic rhinitis			
No OSA	2	0	0.143
Mild OSA	1	0	0.521
Hyperlipidemia			
No OSA	1	1	1.000
Mild OSA	1	0	0.521
Moderate OSA	1	0	0.212
Severe OSA	0	1	0.460
Arthrosis			
Severe OSA	2	0	0.028*
Osteoporosis			
Severe OSA	1	0	0.140

*p value <0.05; *OSA* obstructive sleep apnea

### Oral local risk factors

The local oral risk factors were evaluated in each patient, and it was identified that the average number of teeth present was 25.33 (95% CI (SD 24.08–26.58)) and that the average number of missing teeth was 6.7 (95% CI (SD 5.45–7.94)), but there were no significant differences among the 4 study groups (*p* = 0.1901). Next, the type of oral hygiene according to the O’Leary index was examined, and the findings revealed that it was deficient in 70% of the patients, questionable in 20%, and acceptable in 10%. This distribution is expected since 100% of the population evaluated showed an accumulation of biofilm. The presence of stones was 83.33%, with a predominance rate in the anteroinferior area of 94%. These results indicate that in general, the patients evaluated did not have good oral hygiene habits, and when the relationship between the presence of OSA and periodontitis was evaluated, no statistically significant association was found (*p*> 0.05). On the other hand, when grouping occurred according to all dental malpositions, there was a tendency for patients with OSA and periodontitis to present them more frequently (32.47%); however, no statistically significant association was found between these conditions (*p*> 0.05). In addition to the patients who had open interproximal contacts, 60.8% had periodontitis, but there was no association (*p*> 0.05). In the occlusion analysis, it was found that the percentage of premature contacts was 28.33%, occlusal interferences 45%, wear facets 41.67%, abfractions 23.33%, open bite 6.67%, and edge-to-edge bite 15%.

In the analysis of the relationships between these local risk factors with OSA and the diagnosis of periodontitis, it was evident that there were more patients with severe OSA without periodontitis who used removable partial dentures than patients with severe OSA with periodontitis, and the association was significant (*p* = 0.028). In addition, it was observed that those with severe OSA with periodontitis had more maladaptive resins than those who had severe OSA without periodontitis (*p* = 0.038) (Table 4).

**Table 4:** Frequency and mean of local risk factors associated with periodontal condition and obstructive sleep apnea diagnosis

Local risk factors	Frequency			Mean		
	Without periodontitis (*n*=15)	Periodontitis (*n*=15)	*P* value	Without periodontitis (*n*=15)	Periodontitis (*n*=15)	*P* value
Removable partial denture						
No OSA	1	2	0.543	0.07	0.13	0.5589
Mild OSA	0	1	0.087	0	0.33	0.1039
Moderate OSA	1	0	0.212	0.33	0	0.2856
Severe OSA	2	0	0.028*	0.5	0	0.0273*
Leaking resins						
No OSA	3	3	1.000	0.93	0.2	0.2249
Mild OSA	2	1	0.782	0.75	0.67	0.9418
Moderate OSA	1	2	0.659	1	0.5	0.6039
Severe OSA	0	5	0.038*	0	3.13	0.1729
Buco-distal rotation						
No OSA	3	7	0.121	0.27	1.13	0.0418*
Mild OSA	2	1	0.782	0.25	0.67	0.3893
Moderate OSA	1	3	0.270	1	1.75	0.5922
Severe OSA	2	2	0.386	0.5	0.5	1.000

*p value <0.05; *OSA* obstructive sleep apnea

On the other hand, in the evaluation of the average of local risk factors associated with the periodontal condition and the diagnosis of apnea, it was recorded that patients without OSA with periodontitis on average had more buco-distal rotations than those without OSA without periodontitis (*p* = 0.0418) (Table 4).

## Discussion

The present study was conducted with the objective of determining the relationship of OSA with the periodontal condition and its associated local and systemic risk factors.

When evaluating the association between patients who presented OSA with a periodontal condition, it was found that of those diagnosed with periodontitis, 66.66% presented with severe OSA, showing a tendency for patients with periodontitis to present with severe apnea, although the difference was not significant. The relationship between OSA and the periodontal condition has been reported in recent years, and Gunaratnam et al. [11], in whose study the number of patients was quite similar to that of the present study (66 participants), reported that the prevalence of periodontitis in patients with OSA was four times higher. Likewise, Ahmad et al. [18], in a case-control study with 154 patients (50 cases and 104 controls), reported that cases were 4.1 times more likely to have a high risk of OSA than controls. Similar to the results reported in the present study, Loke et al. [19] found that the prevalence of periodontitis in the OSA group (96.4%) was significantly higher than that in the control group (75%) and that 48.2%% of patients with periodontitis had severe OSA. Consistently, Al-Jewair et al. [20], Keller et al. [21], Sanders et al. [22], and Latorre et al. [23] showed a positive association between OSA and periodontitis. However, there is controversy in this regard, and in contrast to the above, Kale et al. [5], in a study with 260 patients, did not find significant differences between the OSA groups for the presence or absence of periodontitis.

Different hypotheses have been postulated to explain the mechanism by which periodontal disease causes OSA or vice versa. One of them is that the relationship between the two conditions is comorbid rather than causal, since both share common risk factors [11]. Another hypothesis is that periodontitis produces chronic inflammatory responses in susceptible hosts and acts as a mediator of systemic inflammation of OSA or vice versa [8, 11, 20, 24]. It has also been reported that mouth breathing associated with OSA increases the expression of periodontitis, which may occur as a consequence of a greater accumulation of biofilm favoring the colonization of periodontopathogenic microorganisms caused by xerostomia [11, 22, 24]. Finally, it has been proposed that it may be due to oxidative stress that occurs in both pathologies [22, 25].

When analyzing the periodontal variables in slightly more detail, it was reported that patients with periodontitis with and without OSA had a higher percentage of bleeding on probing, biofilm, greater depth of gingival sulcus, periodontal pockets, and a greater loss of clinical attachment level, with statistically significant differences (*p* < 0.05). Scientific evidence repeatedly shows us that bleeding on probing, the percentage of biofilm, the depths of periodontal pockets, and the greatest clinical attachment losses are related parameters and are altered in the presence of periodontitis [7, 17].

In the comparison of patients without periodontitis, the biofilm percentage was higher in patients with OSA. These results are similar to those of Gamsiz-Isik et al. [13], who found that the biofilm index was higher in patients with OSA than in controls. This has been associated with the daytime sleepiness experienced by these patients, which leads to a lower frequency of daily brushing in patients with OSA [26]. It has also been associated with oral breathing during sleep since it favors the accumulation of biofilm on the surface of the teeth and the inability to eliminate it effectively due to the decrease in salivary flow [27].

The average age of the patients was 49.4 years, and that of patients in the OSA group with periodontitis was 53.2 years, similar to the results by Seo et al. [28]. Indeed, there is scientific evidence that aging is positively related to an increase in the incidence of OSA [23, 29]. In fact, a more recent study reported that due to aging, the number of obstruction sites and the pattern of collapse may vary due to changes in the pharyngeal anatomy, redistribution of body fat, and greater laxity of the muscular and hypopharyngeal structures. In their results, they determined that the value of AHI increased with aging [30].

With respect to the systemic risk factors evaluated, it was found that one of the most frequent diseases was obesity, affecting 58.3% of the patients. The literature has suggested that obesity plays a role in the pathogenesis of OSA due to alterations in the structure and function of the upper respiratory tract. Obesity according to Ong et al. [31] and Al-Qahtani et al. [32] also induces an inflammatory state since adipose tissue is an abundant source of proinflammatory cytokines that are associated with defects in neuromuscular control of the respiratory tract that lead to higher susceptibility to and severity of OSA.

On the other hand, in the analysis of obesity according to a study group, it was found that in the group of patients with apnea and periodontitis, the percentage of patients (34%) with obesity was higher. This finding has been previously reported in the literature, where Martinez-Herrera et al. [33] related obesity with periodontitis. Likewise, in a systematic review, Keller et al. [34] determined that overweight, obesity, weight gain, and increased waist circumference can be risk factors for the development of periodontitis and even increased disease severity. It has been shown that obesity is higher in patients with periodontal disease than in periodontally healthy patients [21].

In the evaluation of BMI in the general population, an average of 26.12 kg/m^2^ was found, being higher in patients in the apnea group with periodontitis (28.04 kg/m^2^). Although no significant differences were found among groups, there was a trend showing that patients with OSA tend to have a higher BMI. These results are similar to those reported by Cuervo et al. [35]. Additionally, Martinez-Herrera et al. [33] revealed that both BMI> 35 kg/m^2^ and neck circumference> 40 cm were risk factors for OSA, but after adjustments were made for all other variables, the difference was not significant for BMI but was significant for neck circumference. This may be because BMI is an indicator of total adiposity, but it does not evaluate the distribution of body mass. Therefore, it does not provide specific information related to localized fat deposition around the neck, which can be an accurate predictor of OSA.

Arterial hypertension (AHT) was another of the most common systemic conditions since 28.33% of patients had AHT. Likewise, it was evident that in the group of patients with severe OSA, there were a greater number of patients with AHT. The relationship between OSA and AHT has been reported previously, and in accordance with the results, González-Pliego et al. [36] reported the coexistence between OSA and AHT, relating OSA as one of the most common causes of secondary AHT. Likewise, Latorre et al. [23] and Rimoldi et al. [37] reported an association between AHT and OSA. It is evident then that the vast majority of studies indicate that OSA and hypertension coexist and are common.

The present investigation found that patients without periodontitis had more AHT than patients with periodontitis, with a statistically significant difference (*p* = 0.038). These results are contrary to those reported by Latorre et al. [23] who found a significant association between OSA and chronic periodontitis or AHT, as well as an association with hypertensive cardiomyopathy, in a much larger sample of 190 patients.

Hypothyroidism was the third most common systemic pathology reported since 16.6% of the general population had it. Kuczyński et al. [38] and Thavaraputta et al. [39] also reported an association between OSA and hypothyroidism. In contrast to these results, in a study conducted by Bielicki et al. [40] in 813 patients diagnosed with OSA, 4.7% had hypothyroidism, but there was no significant relationship between these two pathologies.

In the analysis of the associated local factors, a statistically significant association was found between severe OSA and removable partial dentures. This relationship has been studied since the report that edentulism can generate a loss of vertical dimension, reduction of the height of the lower face, and rotation of the mandible, which leads to disharmony of the occlusion causing an alteration of the upper airways [41, 42]. Additionally, it has been found that the tongue could be positioned in a manner that obstructs the airway during sleep [32]. One of the first to explain this association was Bucca et al. [43], who reported that removing the removable prosthesis at night significantly decreases the retropharyngeal space and that sleeping without it is associated with a significant increase in the severity of OSA and a decrease in oxygen saturation.

In the evaluation of the relationship between decayed, lost, and filled teeth in the present study, it was shown that the average number of missing teeth was 6.7, and the percentages of patients with caries and filled teeth were 15% and 79.9%, respectively. A significant association among severe OSA, periodontitis, and maladaptive resins was evident. Additionally, data have shown an association between periodontitis and maladaptive restorations, whether interproximal or cervical, since they are bacterial niches that trigger inflammation and destruction of periodontal tissues [44, 45].

Continuing with local factors, in the analysis of malocclusion, open bite and edge-to-edge bite were evaluated. However, no significant association with OSA was found in this study. These results are consistent with those reported by Alqahtani et al. [46] and Gudipaneni et al. [47] in adult patients. In contrast, associations between anterior open bite, posterior crossbite, lip incompetence, and OSA symptoms have been reported in adolescent patients [47, 48].

When evaluating the facets of dental wear, it was found that 41.67% of the population had them, and despite this high prevalence, there was no significant association with OSA or periodontitis. In contrast, a study conducted by Durán-Cantolla et al. [49] reported a statistically significant correlation between tooth wear and OSA severity and indicated that the prevalence of OSA in patients with tooth wear was three times higher than that in patients in the general population. In the literature, it has been reported that nocturnal bruxism could be a factor associated with tooth wear [50].

## Conclusions

In light of the limitations of this study, it can be concluded that:

- Patients with periodontitis have a higher prevalence of severe OSA.
- Patients with OSA and periodontitis have a tendency toward more obesity and hypothyroidism.
- Patients with OSA have a higher BMI regardless of the diagnosis of periodontitis.
- There is an association between arterial hypertension and severe OSA, as well as between osteoarthritis and severe OSA.
- There is an association between the use of a removable partial denture and severe OSA.
- There is an association among the presence of maladaptive resins, severe OSA, and periodontitis.

## Recommendations

- This work suggests that future research should examine the association between different local and systemic risk factors in which a statistically significant association was evidenced.

## Abbreviations

AASM: American Academy of Sleep Medicine
OSAHS: Obstructive sleep apnea-hypopnea syndrome
OSA: Obstructive sleep apnea
TNF-α: Tumor necrosis factor alpha
IL: Interleukin
NF-Kβ: Nuclear factor kappa beta
RANK-L: Ligand of the nuclear factor activator receptor κB
PGE2: Prostaglandin E2
PCR: C-reactive protein
PSG: Polysomnographies
AHI: Apnea-hypopnea index
BMI: Body mass index
AHT: Arterial hypertension
CAL: Clinical attachment level
